# Social behavioural profiles in pigs and the role of sex, dominance and kinship

**DOI:** 10.1016/j.animal.2026.101807

**Published:** 2026-05

**Authors:** P. Seddaiu, S.P. Turner, V.E. Lee, M. Brims, I. Camerlink

**Affiliations:** a Animal Behaviour and Welfare Department, Institute of Genetics and Animal Biotechnology of the Polish Academy of Sciences, Postępu 36, 05-552 Jastrzębiec, Poland; b Animal Behaviour & Welfare, School of Veterinary Medicine and Biosciences, Scotland’s Rural College (SRUC), West Mains Rd., Edinburgh EH9 3JG, UK

**Keywords:** Affiliative, Glicko rating, Principal component analysis, Proximity, *Sus scrofa*

## Abstract

Affiliative behaviours in pigs can enhance group cohesion and lower stress levels, ultimately improving individual welfare. Individual factors such as dominance rank, sex or kinship may play a key role in shaping the expression of social behaviours, but there is a lack of knowledge on the contribution of these variables. The aim of this study was to identify social behavioural profiles in pigs, based on putative affiliative behaviours, and evaluate the extent to which dominance rank, sex, and kinship influence their expression. Agonistic interactions were recorded on 212 male and female domestic pigs (*Sus scrofa domesticus*) to calculate dominance ranks within 16 groups. Based on this, 96 pigs (six pigs per group) were selected for observations on detailed social nosing behaviours, allogrooming, spatial proximity and social play. Principal components analysis was used to assess the presence of behavioural profiles, followed by mixed model analysis to evaluate the influence of individual factors on each principal component (**PC**). Snout contact constituted the majority of interactions and was exhibited by all pigs. Lying in body contact and snout–snout proximity were also frequent and were expressed by more than 95% of individuals. Allogrooming and social play were observed in 29.2 and 33.3% of pigs, respectively, and represented less than 1% of the total interaction frequency. Three PCs had eigenvalues > 1 and together explained 60.9% of the variance. The PCs related to social contact (**PC1**), proximity (**PC2**) and social engagement (**PC3**). Sex, dominance status and kinship had no effect on PC1 or PC3, but sex and kinship had a limited effect on PC2, with entire males showing more snout proximity than females (*P* < 0.001) and pigs showing less snout proximity behaviours towards their kin (*P* = 0.044). This study shows that the expression of putatively affiliative social behaviours can be clustered into profiles and is under commercial settings only marginally influenced by individual factors such as dominance and kinship, suggesting their general relevance to pigs’ social life.

## Highlights

• This study investigated factors influencing social behaviour in pigs.
• Three behavioural profiles identified: social contact, proximity, and social engagement.
• Entire males engaged more frequently in snout−proximity behaviours than females.
• Pigs showed less snout−proximity behaviours towards their kin.
• Recognising social variation may improve pig welfare and group management.

## Implications

This study examined physical contact and proximity behaviours in group-housed pigs. Pigs differed in their social behavioural profiles, expressing varying degrees of social contact, proximity, and social engagement. This suggests that social behaviour in pigs is flexible rather than fixed into discrete types. In commercial environments where pigs are housed in groups, recognising such individual variation may help explain differences in social dynamics and group cohesion. The observed sex difference in proximity behaviours further indicates that management strategies may need to consider sex-specific social tendencies. Overall, these findings highlight the importance of accounting for individual social variation in farmed pigs.

## Introduction

Social behaviours are crucial to the development of animals, particularly in highly social species (Snyder-Mackler et al., 2020, Lee et al., 2022). Among these, affiliative behaviours — those that promote social bonding and cohesion — play a key role in maintaining group stability, reducing stress, and supporting individual welfare (Cameron et al., 2009, Silk et al., 2010, Campbell et al., 2018). In farm animals such as domestic pigs (*Sus scrofa*), these interactions have been linked to better welfare (Val-Laillet et al., 2009, Rault, 2019). Affiliative behaviours are also commonly shown in wild animals, where they may play an important role in maintaining social bonds that are adaptive and benefit fitness (Silk et al., 2010, Snyder-Mackler et al., 2020). Despite the importance of affiliative behaviour, it has received much less research interest in farm animals than agonistic or competitive behaviour (Boissy et al., 2007, Rault, 2012). Understanding the dynamics of affiliative behaviours in farm animals is therefore of fundamental importance, especially in the context of animal husbandry, where group composition is often manipulated.

In pigs, affiliative behaviours have been described in a variety of contexts, including postconflict affiliation (Cordoni et al. 2023), social cohesion (Camerlink et al., 2014), and stress buffering (Reimert et al., 2014, Khatiwada et al., 2025a). Several behaviours may be considered as putatively affiliative due to their occurrence across various contexts, including agonistic events, indicating that these behaviours may not always be purely affiliative, and their function can depend on the social context (Goumon et al., 2025). Such behaviours include allogrooming (Meynhardt, 1982, Seddaiu et al., 2025), resting in body contact (Newberry and Swanson, 2001, Seddaiu et al., 2024), social play (Horback, 2014, Brown et al., 2015, Steinerová et al., 2024) and snout contact (Camerlink and Turner, 2013, Khatiwada et al., 2025b). The nature and frequency of the aforementioned behaviours, as well as the factors that influence them, are still under investigation.

Recent studies have shown that behaviours such as snout contact and lying in body contact can depend on external conditions such as ambient temperature (Camerlink et al., 2022a) and group stability (Khatiwada et al., 2025b). However, the influence of individual factors on the expression of affiliative interaction, such as sex, genetic relatedness and dominance status, is not yet clear. Two studies on groups of growing pigs reported that sex, dominance status and genetic relatedness were only partially related to potentially affiliative behaviours (Goumon et al., 2020, Clouard et al., 2024). However, other studies have shown that there is an effect of dominance status on spatial proximity in pigs, with dominant pigs often spacing further away from others, eventually resulting in less social interactions initiated (McCort and Graves, 1982). Moreover, a sex effect has been seen in conflict avoidance behaviours, where entire males often engaged in ritualised interactions (i.e. proximity-based interactions) before escalating into fights, whereas females attacked sooner (Camerlink et al., 2022b). Sex differences have also been reported in social nosing interactions among piglets (Clouard et al., 2022). Further, familiarity might influence the expression of a range of social behaviours, with familiar pigs nearly twice as likely to express snout contact (Jowett et al., 2025). Despite growing interest in affiliative behaviours in farm animals (Goumon et al., 2025), there is a limited and contradictory understanding of how these individual factors interact to shape their expression.

The aim of this study was to identify behavioural profiles in pigs based on putatively affiliative behaviours and evaluate the extent to which dominance rank, sex, and kinship influence their expression. While most recent studies on pig sociality have focused on single behaviours, these provide only a partial view of the complexity of social life. In contrast, this study takes a wider lens, analysing how behaviours can co-occur to form distinct behavioural profiles. Based on the existing literature, we hypothesised that genetically related individuals would show more social interactions, that males would display more frequent proximity-related behaviours than females, and that dominant individuals would initiate fewer social interactions than mid− or low−ranked individuals, after accounting for the effect of sex.

## Material and methods

### Animals and housing

In total, 16 groups of domestic pigs (Large White × Landrace sows × American Hampshire boars) were studied in the Easter Howgate Pig Research Unit from August 2024 to March 2025. A total of 212 pigs (n = 108 entire males and n = 104 females) from three farrowing batches were observed for dominance rank. Each batch was observed in a different month. From these, 96 pigs (47 males and 49 females) were further observed as focal animals (described below). Cross-fostering was kept to a minimum and done only for litters with more than 10 piglets and where the sow had a body condition below the herd average. Pigs were weaned at 4 weeks and regrouped into single-sex groups. At 8 weeks of age, pigs were regrouped into new single sex groups of 10–16 individuals each. While mixing events are known to influence social behaviour in pigs (Petherick and Blackshaw, 1987), all individuals experienced the same mixing history, allowing behavioural variation to be examined under comparable social conditions. The study started after this second event of mixing at 8 weeks of age and finished at 12 weeks of age. Pigs were grouped by similar weight and siblings were divided over as many pens as possible, whilst ensuring that each pig had at least one sibling and one pig from the same weaning pen in their new group. Female pigs were housed in pens that previously housed males and vice versa, so that no pig remained in its original pen, to avoid the effect of home pen territory on aggression. The groups were housed in enriched conditions with a floor space of 6.9 × 2.1 m (14.49 m^2^; 1.45–0.91 m^2^/pig). Straw was provided as bedding material, with dirty straw removed and fresh straw added as necessary each day. One Porcichew toy (Ketchum manufacturing company, UK) and one length of Manila rope (Westward Rope and Wire, UK) hung from the pen wall were provided as enrichment objects. Shed temperature was adjusted according to the age of pigs and controlled automatically. Food and water were provided *ad libitum*. Pigs were individually recognised by their ear tag and by a spray mark applied on their back with animal marker spray (East Riding Farm Services, UK). The back mark for identification was redone twice a week, in the morning, at least 1 h before starting the observations. Daily checks were done to evaluate the pigs’ health.

### Dominance rank analysis

For the first week of observations, after regrouping at week 8, only agonistic behaviours were scored with the aim to calculate dominance relationships. In particular, submissive behaviour was scored to assess who lost the encounter. Submissive behaviour was scored as withdrawal during an agonistic encounter, including a head-tilt-retreat, whereby the losing pig lowers its head and turns it rapidly sideways (Jensen, 1980), thereby turning the shoulder away from the opponent and refraining from retaliation for at least 10 s (Camerlink et al., 2016). Agonistic interactions where it was unclear whether a head-tilt-retreat had occurred were recorded as inconclusive. Pigs were observed by continuous observations for a total of 5 h per pen through live and video recordings between 0900 and 1600 h. Subsequently, a dominance score was determined for each individual using the Glicko rating from the agonistic interactions with a clear outcome. Glicko rating is a system for establishing which individual is dominant based on the outcomes of their fights. The system assigns a greater increase in score to an individual when it defeats a highly successful individual than a less successful individual. It incorporates a rating value and a rating deviation (**RD**), which quantifies the certainty of the rating (Glickman, 1999). The model is particularly well-suited for datasets with varying numbers of interactions per individual and allowed us to determine a dominance rank from the individual scores. Glicko ratings were calculated in R software version 4.5.1 (R Core Team, 2025), using the “PlayerRatings” package (Stephenson and Sonas, 2012). After the analysis, each pig had a final rating value and a final RD based firstly on the number of victories and defeats, and also on the relative dominance exhibited during each interaction. The final ranking was determined by both the rating value and the associated RD, reflecting not only performance outcomes but also the confidence in those estimates. Default values were set at 2 200 for the rating and 300 for the RD.

### Behavioural observations

Based on the Glicko ratings, six focal pigs were selected per group. For each group, two pigs with the highest, the middle and the lowest ranks based on their Glicko scores were selected for further observations. The RD was not used in the selection of focal pigs. This resulted in 96 pigs (males and females) being included in the study. Observing six focal pigs instead of 10–16 allowed for more manageable and thus more detailed observations, leading to more accurate and reliable results. Additionally, by selecting from across the dominance hierarchy, a balanced representation within each group was maintained. The focal animals were observed in weeks 10 and 11 using the ethogram described in Table 1, using continuous observations. All interactions were scored as single events and attributed to one actor, meaning that in the case of repeated events in a short time, every contact was scored. For each interaction, the actor was defined as the individual who initiated the behaviour, regardless of whether the recipient was a focal or non-focal pig. Interactions between two focal pigs were recorded once, attributed only to the initiating individual. In case of behaviours that could appear symmetrical, the actor was identified as the pig that initiated the approach leading to the interaction. If no clear initiator could be identified, the interaction was not scored. Pigs were observed for 10 days over 2 weeks, 5 days a week, for a total of 10 h of continuous observation per pen, resulting in 160 h in total. All observations were performed using the Animal Behaviour Pro App, version 1.2 (University of Kent, Canterbury, UK), installed on an iPad.

**Table 1:** Ethogram for observed behaviours in 96 growing pigs of 10–11 weeks of age.

Behaviour	Description
Snout-snout contact	Touches another pig’s snout with its nose disc; includes the nose disc of the recipient
Snout-snout proximity	A pig extends its snout in direction of the snout of another pig snout at approximately <30 cm distance without making contact
Snout-head contact	Touches another pig’s head with its nose disc; excludes the snout and allogrooming
Snout-head proximity	A pig extends its snout in the direction of the head of another pig at <30 cm distance without making contact; excludes snout
Snout-body contact	Touches the body of another pig with its nose disc, excluding the head
Allogrooming	Gently nibbles or licks the body or head of another pig; does not cause acute skin damage to the other pig
Lying in body contact head-to-head	Focal pig actively lies down with its body in contact with a conspecific in a parallel orientation with their heads adjacent to or touching
Lying in body contact any other orientation	Focal pig actively lies down with its body in contact with a conspecific in a parallel head-to-tail, or non-parallel orientation
Social play	A playful interaction characterised by one or more of the following behaviours: running (incl. chasing), hopping, flopping, tossing, pushing or nudging without delivering or receiving potentially damaging aggression. It involves at least one other individual besides the actor.

### Statistical analysis

For each pig, we analysed the total frequency of initiated interactions for each behaviour and based on that constructed a frequency matrix in Excel. The descriptive statistics were conducted in Excel 2019 and the Principal Component Analysis (**PCA**) and mixed models in R version 4.5.1. As descriptive analysis, for each behavioural variable, we calculated the total number of occurrences, the average frequency per individual, the percentage of individuals expressing the behaviour at least once, and the percentage of each behaviour expression relative to the total (Table 2).

**Table 2:** Frequency distribution of observed social behaviours. Columns show total occurrences across all animals, average frequency per pig, percentage of pigs expressing the behaviour, and percentage of each behaviour expression relative to total. A total of 7 097 interactions were scored during 160 h of observations from 96 pigs (16 groups) aged 10–11 weeks.

Behaviour	Total occurrences	Average frequency per pig	% of pigs performing behaviour	% of total interactions
Snout-body contact	1 925	20.1	100%	27.1%
Snout-snout contact	1 628	17.0	100%	22.9%
Snout-head contact	1 186	12.4	100%	16.7%
Lying in body contact any other orientation	775	8.1	99.0%	10.9%
Lying in body contact head-to-head	634	6.6	97.9%	8.9%
Snout-snout proximity	602	6.3	96.9%	8.5%
Snout-head proximity	253	2.6	85.4%	3.6%
Allogrooming	44	0.5	29.2%	0.6%
Social play	50	0.5	33.3%	0.7%

#### Principal component analysis

The first aim of our analysis was to identify behavioural profiles in our sample with PCA. This approach reduces data dimensionality by transforming the original variables into a new set of uncorrelated variables (principal components, **PCs**) that capture most of the dataset’s variance (Abdi and Williams, 2010). Each PC is characterised by a set of co-occurring behaviours and can therefore be interpreted as a behavioural profile. Individual PC scores reflect the extent to which each animal expressed that profile. All behavioural variables were available for all focal individuals. Zero values reflected the absence of a behaviour during the observation period rather than missing observations; therefore, no data removal or imputation was required prior to the PCA. Before extracting the principal components, the data were standardised (argument scale = TRUE in the prcomp function) using z-transformation (mean = 0, SD = 1) and inspected for outliers using boxplots, with observations identified as outliers if they had a z-value greater than 3, following Tabachnick et al. (2007). A small number of outliers were identified (12 data points out of 864 behavioural measurements across 11 individuals); however, sensitivity analyses excluding these individuals produced a comparable PCA structure. Results from the full dataset were therefore considered. We then assessed the suitability of the dataset for PCA analysis. Pearson correlations were calculated between all z-transformed variables. The Kaiser-Meyer-Olkin measure of sampling adequacy was 0.73, and Bartlett’s test of sphericity was significant (χ^2^ = 171.86, *P* < 0.001), indicating that the correlation structure of the behavioural variables was suitable for PCA. We performed a PCA with orthogonal rotation using the prcomp function from “stats” package to run the analysis. We used the “factoextra” package (Kassambara and Mundt, 2016) to visualise the results with graphs. Once all principal components were extracted, only those with eigenvalues exceeding 1 were retained for further analysis, following the Kaiser criterion (Kaiser, 1960).

#### Mixed models

Once the principal components were identified, we aimed to assess the influence of dominance rank, sex, and kinship on the expression of each component. To do so, we extracted the PC scores for each individual across all three components. These scores were then used as response variables in linear mixed models, using the lmer function from the “lme4” package in R (Bates et al., 2024). Sex (male vs. female), dominance score (high, mid, low), and kinship-based interactions (proportion) were included as fixed effects. Variables pen and batch, which also accounted for seasonal fluctuations, were added as random effects. Model assumptions were assessed by inspecting the residuals. Histograms and QQ-plots indicated approximate normality, and residuals versus fitted values plots confirmed homoscedasticity. Multicollinearity among fixed effects was evaluated using variance inflation factors (**VIFs**), with all VIFs well below 5 (dominance: 1.00, kinship-based interactions: 1.01, sex: 1.00), indicating no collinearity among fixed effects. *P-*values < 0.05 were considered significant. For the kinship-based interactions, because the number of siblings varied between groups, as well as the total number of group members, we corrected kin interaction rates according to the opportunity pigs had of interacting with a sibling. For each focal pig and behaviour, we calculated an adjusted kinship proportion, defined as:

$$\mathrm{Adjusted}\;kinship\;proportion=\frac{N\;interactions\;with\;kin/N\;total\;interactions}{N\;siblings\;in\;a\;group/N\;individuals\;in\;a\;group-1}$$

This formula calculates whether individuals interacted with kin more or less than expected based on availability and is derived from the simple ratio index from Whitehead and James (2015). Values > 1 indicate a bias towards kin, values = 1 indicate interactions proportional to availability, and values < 1 indicate kin avoidance.

## Results

### Dominance ranks and behavioural expression

Glicko rating values, reflecting dominance ranks, ranged from 1 433 to 2 970 across all 212 individuals (2 189 ± 314.8 SD). The rating deviation, which quantifies the certainty of the rating, ranged from 106.1 to 237.2 (149.8 ± 22.88 SD). Pigs who consistently won encounters exhibited higher ratings and lower RD values. In contrast, individuals with fewer interactions or inconsistent win-loss outcomes had higher RD values, reflecting increased uncertainty. Before conducting the PCA, we examined the frequency of each behaviour (Table 2). The majority of interactions consisted of nosing contacts. Behaviours such as lying in body contact and snout–snout proximity were also common and were expressed by more than 95% of individuals. In contrast, low-frequency behaviours such as allogrooming and social play were observed in 29.2 and 33.3% of pigs, respectively. Although these behaviours occurred less frequently overall (< 1% of total interactions), they still contributed to individual variation and were therefore retained in the PCA.

### Behavioural profiles

The PCA on the social behaviours of 96 focal pigs identified nine components with distinct loading profiles. However, only three principal components (**PC1**, **PC2**, and **PC3**) had eigenvalues greater than 1 and were therefore selected for further analysis. These three components combined explained a total of 60.9% of the variance among the behavioural variables (Table 3). PC1 presented strong positive loadings (≥0.5) on the frequency of making snout contact with the snout or the head of another pig, as well as on lying in body contact with a conspecific (head-to-head or in any other orientation). Behaviours belonging to PC1 were the most frequent, constituting a minimum of 8.9% and a maximum of 27.1% of the total recorded frequencies. Furthermore, almost all the individuals expressed these behaviours (97%−100%) (Table 2). PC2 showed high positive loadings on proximity behaviours (snout-to-snout and snout-to-head). These behaviours accounted for 8.5 and 3.6% of all behavioural occurrences and were expressed by 96.9 and 85.4% of individuals (Table 2). Consequently, they were less common than the behaviours that loaded most strongly onto PC1. PC3 exhibited high positive loadings on allogrooming and social play. PC3 behaviours occurred much less frequently (allogrooming: 0.6% and social play: 0.7%) and were expressed by only 29.2 and 33.3% of individuals (Table 2). Based on these behavioural associations, the components were labelled as follows: PC1 - social contact, PC2 - proximity, and PC3 - social engagement.

**Table 3:** Principal component analysis (PCA) loading scores of behaviour measurements on principal component 1 (PC1), principal component 2 (PC2) and principal component 3 (PC3). Based on social interactions observed in 96 pigs (16 groups) of 10–11 weeks of age.

Behaviour	PC1	PC2	PC3
Snout-snout contact	0.782	−0.018	0.168
Snout-snout proximity	0.059	0.851	0.080
Snout-head contact	0.815	0.104	0.096
Snout-head proximity	0.135	0.808	−0.061
Snout-body contact	0.566	0.188	0.068
Allogrooming	0.023	0.095	0.904
Lying in body contact head-to-head	0.688	−0.038	0.096
Lying in body contact any other orientation	0.742	−0.021	−0.152
Social play	0.228	−0.441	0.503
Variance explained	31%	18.2%	11.7%
Eigenvalue	2.791	1.636	1.053

### The role of sex, dominance rank and kinship

**Fig. 1:**
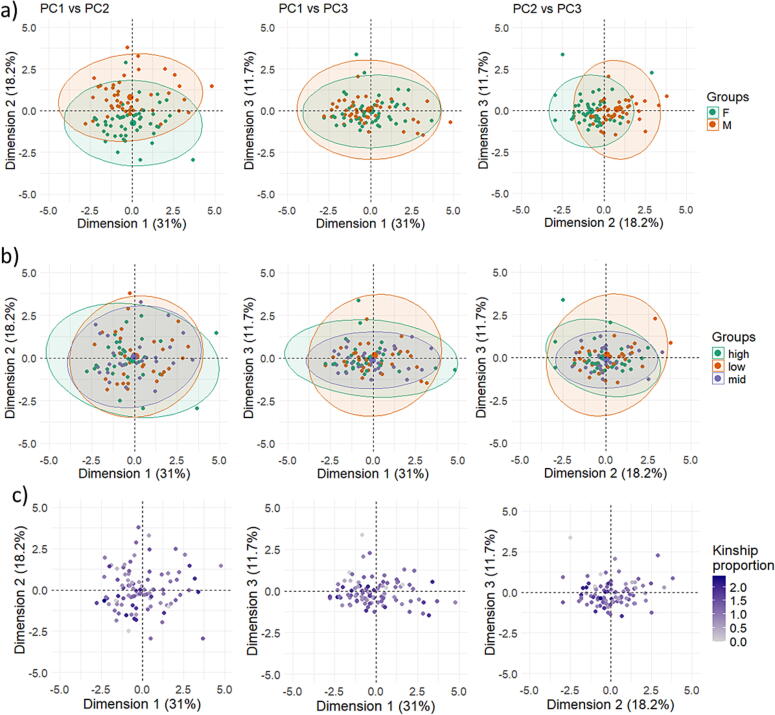
Principal Component Analysis (PCA) scatterplots showing the distribution of individual pigs along the first three principal components. Each plot shows a combination of the first three principal components (PCs), with PCs and their variance on x- or y-axis. Each point represents one individual (n = 96 pigs). (a) PCA score plots grouped by sex (female (F), male (M)) with 95% confidence ellipses. (b) PCA score plots grouped by dominance status (high, mid, low), with 95% confidence ellipses. (c) PCA score plots coloured by kinship proportion within the social group, where darker shades indicate a higher proportion of kin interactions.

**Fig. 2:**
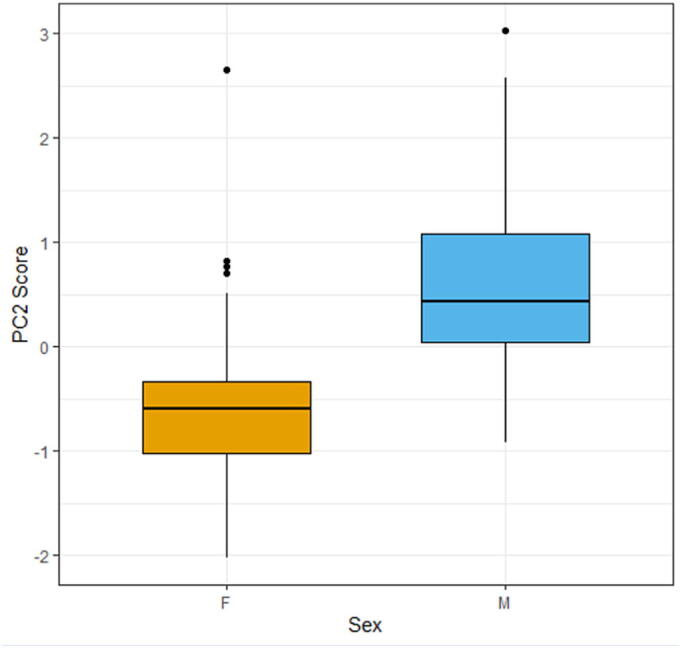
(a) Boxplot of the distribution of principal component 2 (PC2) scores (y-axis) for female (F) and male (M) pigs (x-axis). The boxes represent the interquartile range (IQR), which contains the middle 50% of the data. The horizontal line inside the boxes represents the median. The vertical lines extending from the box (whiskers) reach the minimum and maximum values, excluding outliers.

In the mixed model analysis, sex, dominance rank and kinship did not significantly influence PC1 ‘social contact’ or PC3 ‘social engagement’ (Fig. 1). However, sex influenced PC2 ‘proximity’ (estimate: 1.17 ± 0.16 SE, *P* < 0.001), indicating that males engaged more frequently in the proximity behaviours with higher loadings on this component than females (Fig. 2). Kinship had a limited effect on PC2 scores (estimate: −0.36 ± 0.18 SE, *P* = 0.044), indicating that higher relatedness was associated with lower PC2 values.

## Discussion

This study explored how domestic pigs form behavioural profiles based on putatively affiliative behaviours and to what extent the behavioural profiles are shaped by individual factors. We expected that individuals would exhibit behavioural profiles, each characterised by different combinations of behaviours. Principal Components were interpreted to represent ‘social contact’, ‘proximity’ and ‘social engagement’ and together explained 60.9% of the variation. We hypothesised that subordinate pigs would initiate fewer social interactions, for which we found no evidence. However, the hypothesis that males would display more frequent proximity-related behaviours than females was indeed confirmed, based on PC2. Against expectation, kinship did have a limited effect on social tendencies.

From the PCA, three clear PCs were extracted. PC1 (social contact) was characterised by high loadings of behaviours involving direct physical contact, such as snout contact and lying in body contact. PC1 explained the greatest percentage of variance among all components (31%) and included the most frequent behaviours. Snout contact and lying in contact have been frequently used in studies on affiliative behaviour in pigs (Goumon et al., 2025), but most studies so far suggest that these two behaviours are distinctly different and may represent separate facets (Camerlink et al., 2022a, Clouard et al., 2024). Snout contact serves various social functions, including affiliative contact, whereas lying in body contact has, in recent studies, been shown to be less strongly associated with the maintenance of affiliative relationships (Goumon et al., 2025). Snout contact and lying in contact may occur as part of short behavioural sequences, such as social nosing before approaching a lying recipient (Camerlink and Turner, 2013). This interpretation also helps explain the covariation highlighted by the PCA. As an exploratory and descriptive method, PCA identifies patterns of behavioural co-occurrence and does not imply a biological function. Nevertheless, because these behaviours were the most frequently expressed and involved nearly all individuals, they may have a central role in the social behaviour of pigs. Moreover, the use of interaction frequency rather than duration makes the two contact behaviours seem more similar (being both the most frequently expressed and involving nearly all individuals). In the present study, frequency-based measures were considered more appropriate than duration-based measures to address the objective, as they more directly reflect individual decisions to initiate social interactions. The mixed model analysis showed that dominance rank, sex, and kinship did not significantly influence PC1 scores, indicating that variation in social contact behaviours was not strongly related to these factors. It may indicate that physical contact is of general relevance to social life in pigs (Seddaiu et al., 2024), and it is not strictly related to individual factors.

PC2 (proximity) was characterised by high loadings of snout proximity and explained 18% of the variance among all components. The finding that ‘snout contact’ (PC1) and ‘proximity’ (PC2) emerged as separate components is in line with recent literature that shows that these two similar behaviours (snout contact and snout proximity) have a subtle but distinct meaning for pigs (Khatiwada et al., 2025b, Jowett et al., 2025). Snout proximity has previously been associated with social recognition (Kristensen et al., 2001), which may switch to conflict avoidance and risk aversion during social instability (Khatiwada et al., 2025b). The distinction between snout proximity and snout contact even extends to an inverse relationship with growth, where snout contact relates to BW gain and snout proximity to weight loss (Khatiwada et al., 2025b). Males had a substantially higher score on this PC than females, showing that they are involved in more proximity-related behaviours. Prepubertal entire males were observed to show more proximity-related behaviours than females during agonistic interactions (Camerlink et al., 2022b), which was related to conflict avoidance behaviour. The snout proximity during such ritualised agonistic interactions has greater risks involved than during regular interactions (Camerlink et al., 2022b) and may facilitate conflict avoidance by allowing social assessment without direct physical contact (Khatiwada et al., 2025b). In addition, pigs showed less snout proximity towards their kin, consistent with the idea that snout proximity might serve as recognition and information gathering, while limiting potentially risky interactions with close kin. The effect of sex and kinship on proximity-related behaviours highlights subtle differences in individual sociality that may help refine management practices aimed at improving welfare. For example, increased space allowance (Chidgey, 2024) and barriers to hide behind (McGlone and Curtis, 1985, Bulens et al., 2017) could facilitate the expression of proximity behaviours. While these strategies may be particularly beneficial to entire males, they may also benefit females, accommodating individual variation across pigs. From an evolutionary perspective, these behavioural profiles may reflect pigs’ social competence and behavioural flexibility, which play a key role in adaptation (Taborsky and Oliveira, 2012, Kappeler et al., 2019).

PC3, labelled as social engagement, explained 11.9% of the total variance. It was uniquely composed by allogrooming and social play. Social play in juveniles or adults and allogrooming are infrequently occurring behaviours, which were shown in this study as well as in previous studies (social play: Newberry et al., 1988, Zupan et al., 2019; allogrooming: Seddaiu et al., 2024). Aside from their infrequent occurrence, there are strong individual differences in their expression (play: Brown et al., 2015), with some pigs rarely showing these behaviours, whereas others perform them more frequently (Seddaiu et al., 2024). We did not find an effect of individual factors on PC3. This was unexpected as previous literature indicates that play is shown more by males (Brown et al., 2015, Weller et al., 2019). The reason for this absence might be the combination of allogrooming and social play in the same component. In addition, both Brown et al. (2015) and Weller et al. (2019) studied mixed-sex groups (preweaning stage), whereas in the present study, pigs were housed in single-sex groups. This housing condition might have reduced sex differences in social play frequency. Moreover, social play in pigs is generally inclusive, allowing multiple group members to join (Horback, 2014). This suggests that pigs who initiate social play or give play invites are more socially engaged, independently of internal factors. Furthermore, while allogrooming would be assumed to occur between closely related individuals or those with a high degree of familiarity, there is so far no evidence for this (Clouard et al., 2024).

The dominance status of each individual was assessed through Glicko rating, with higher ratings reflecting more consistent success in agonistic encounters and an ability to defeat other successful animals. The Glicko rating provided a useful method to reflect more precisely the agonistic interactions within the group, while taking into account the opponent’s prior wins and defeats (Glickman, 1999). Domestic pigs establish a relatively stable social hierarchy (through dominance relationships) (Meese and Ewbank, 1973), which is partly based on strength as a result of BW (Lanthony et al., 2022). The Glicko rating did provide a linear hierarchy structure from which we selected the top, mid− and bottom−ranked animals. Nevertheless, their dominance rank did not influence any of the PCs for behaviour, which is in contrast with former studies showing that dominant pigs space further away and may initiate fewer social interactions (McCort and Graves, 1982). Although dominance hierarchies in pigs are typically established within approximately the first 24 h after mixing (Meese and Ewbank, 1973), aggression can continue in the long term due to competition for space and access to resources (Tan et al., 1991). As groups were regrouped only 2 weeks prior, it may be that the hierarchy was still unstable, resulting in a lack of effect of dominance on the PCs. Another explanation is that resources were abundant, reducing the need to enforce dominance hierarchies. It may also be that these social behaviours are expressed regardless of social rank.

## Conclusions

Observations of putative affiliative behaviours on 96 domestic pigs highlighted the presence of three affiliative behavioural profiles: social contact, proximity and social engagement. The profiles were partially influenced by individual characteristics of dominance rank, sex and the relative frequency of kinship-based interactions. The study highlights the difference between behaviours of direct physical contact and those performed in close social proximity without making contact. Together, the results emphasise the social complexity of pigs’ behaviour repertoire, especially for nuanced micro-behaviours related to affiliative interactions.

## Ethics approval

The study was part of a larger overarching research project on social competence in pigs. The study protocol was approved by SRUC’s Animal Experiments Committee. This non-invasive behavioural study was part of a larger experiment authorised by the UK Home Office (Project licence P3850A80D/PP1403242).

## Data and model availability statement

Data are available online under CC BY 4.0 licence at https://doi.org/10.17632/r2svdf6zyh.1. Information can be made available from the authors upon request.

## Declaration of generative AI and AI-assisted technologies in the writing process

During the preparation of this work the author(s) did not use any AI and AI-assisted technologies.

## Author ORCIDs

**Piero Seddaiu:** https://orcid.org/0000-0003-3585-8341.

**Simon P. Turner:** https://orcid.org/0000-0001-9198-9448.

**Victoria E. Lee:** https://orcid.org/0000-0003-3981-387X.

**Mark Brims:** https://orcid.org/0009-0002-4430-0577.

**Irene Camerlink:** https://orcid.org/0000-0002-3427-2210.

## CRediT authorship contribution statement

**P. Seddaiu:** Writing – review & editing, Writing – original draft, Visualisation, Methodology, Investigation, Formal analysis. **S.P. Turner:** Writing – review & editing, Supervision, Project administration, Methodology, Funding acquisition. **V.E. Lee:** Writing – review & editing, Methodology. **M. Brims:** Writing – review & editing, Methodology. **I. Camerlink:** Writing – review & editing, Supervision, Project administration, Methodology, Funding acquisition.

## Declaration of interest

The authors declare that they have no known competing financial interests or personal relationships that could have appeared to influence the work reported in this paper.
